# Leveraging automated time-lapse microscopy coupled with deep learning to automate colony forming assay

**DOI:** 10.3389/fonc.2025.1520972

**Published:** 2025-02-19

**Authors:** Anusha Klett, Dennis Raith, Paula Silvestrini, Matías Stingl, Jonas Bermeitinger, Avani Sapre, Martin Condor, Roman Melachrinos, Mira Kusterer, Alexandra Brand, Guido Pisani, Evelyn Ullrich, Marie Follo, Jesús Duque-Afonso, Roland Mertelsmann

**Affiliations:** ^1^ Collaborative Research Institute Intelligent Oncology (CRIION), Freiburg, Germany; ^2^ Mertelsmann Foundation, Freiburg, Germany; ^3^ Neurorobotics Lab, Department of Computer Science, University of Freiburg, Freiburg, Germany; ^4^ Department of Applied Computer Sciences, LABMaiTE GmbH, Freiburg, Germany; ^5^ Laboratory of Applied Cellular and Molecular Biology, Institute of Veterinary Sciences of the Litoral (ICIVET), Universidad Nacional del Litoral (UNL) - National Scientific and Technical Research Council (CONICET), Esperanza, Argentina; ^6^ Department of Medicine I, Medical Center - University of Freiburg, Faculty of Medicine, University of Freiburg, Freiburg, Germany; ^7^ Department of Pediatrics, Experimental Immunology and Cell Therapy, Goethe University, Frankfurt am Main, Frankfurt, Germany; ^8^ German Cancer Consortium Deutsches Konsortium für Translationale Krebsforschung (DKTK) and German Cancer Research Center (DKFZ), Partner Site Frankfurt, Frankfurt, Germany; ^9^ Frankfurt Cancer Center (FCI), Frankfurt, Germany; ^10^ Lighthouse Core Facility, Medical Center - University of Freiburg, Faculty of Medicine, University of Freiburg, Freiburg, Germany

**Keywords:** automated colony forming assay, time-lapse microscopy, primary B-ALL cells, artificial intelligence, personalized cancer therapy, live cell imaging

## Abstract

**Introduction:**

The colony forming assay (CFA) stands as a cornerstone technique for evaluating the clonal expansion ability of single cancer cells and is crucial for assessing drug efficacy. However, traditional CFAs rely on labor-intensive, endpoint manual counting, offering limited insights into the dynamic effects of treatment. To overcome these limitations, we developed an Artificial Intelligence (AI)-assisted automated CFA combining time-lapse microscopy for real-time tracking of colony formation.

**Methods:**

Using B-acute lymphoblastic leukemia (B-ALL) cells from an E2A-PBX1 mouse model, we cultured them in a collagen-based 3D matrix with cytokines under static conditions in a low volume (60 µl) culture vessel and validated its comparability to methylcellulose-based media. No significant differences in final colony count or plating efficiency were observed. Our automated platform utilizes a deep learning and multi-object tracking approach for colony counting. Brightfield images were used to train a YOLOv8 object detection network, achieving a mAP50 score of 86% for identifying single cells, clusters, and colonies, and 97% accuracy for Z-stack colony identification with a multi-object tracking algorithm. The detection model accurately identified the majority of objects in the dataset.

**Results:**

This AI-assisted CFA was successfully applied for density optimization, enabling the determination of seeding densities that maximize plating efficiency (PE), and for IC50 determination, offering an efficient, less labor-intensive method for testing drug concentrations. In conclusion, our novel AI-assisted automated colony counting platform enables automated, high-throughput analysis of colony dynamics, significantly reducing labor and increasing accuracy. Furthermore, it allows detailed, long-term studies of cell-cell interactions and treatment responses using live-cell imaging and AI-assisted cell tracking.

**Discussion:**

Future integration with a perfusion-based drug screening system promises to enhance personalized cancer therapy by optimizing broad drug screening approaches and enabling real-time evaluation of therapeutic efficacy.

## Highlights

- The study introduces an AI-assisted, automated CFA that integrates time-lapse microscopy-based drug screening for dynamic, real-time insights into treatment effects.
- The AI model, trained on brightfield images, achieved high accuracy in colony identification, with promising results for real-time analysis of B-ALL cells.
- This innovative approach optimizes drug screening processes and supports personalized cancer therapy development.

## 1 Introduction

A colony-forming assay (CFA) is a type of cell survival assay that measures the ability of a single cancer cell to grow into a colony and have an unlimited ability to expand. This proliferative capacity of cancer cells can be used to identify the potential of the cell to form cancer and relapse (1, 2). Hematopoietic cancer cells, such as leukemia cells, exhibit diverse growth properties *in vitro*. These cells can grow in either liquid culture or semi-solid media depending on their characteristics. Healthy hematopoietic progenitor cells, in contrast, can grow in semi-solid media enriched with cytokines but do not proliferate in liquid culture. Similarly, primary leukemia cells from patients typically grow in semi-solid media, although in rare cases, they may also grow in liquid culture (3). Creating a microenvironment that promotes self-renewal without inducing differentiation is also difficult. The precise combination and concentrations of cytokines are critical but hard to optimize. Moreover, the natural bone marrow microenvironment is complex, involving stromal cells, extracellular matrix components, and signaling molecules. Reproducing this *in vitro* is challenging. Therefore, the choice of culture medium and supplements is crucial in the expansion of these cells *ex-vivo* (4, 5). Traditional CFA is performed in a very labor-intensive manual way where the cells are seeded in a 6-well plate embedded in a semi-solid medium such as methylcellulose supplemented with necessary cytokines and nutrients. One of the main applications of CFA is to perform screening of potential drugs with anti-tumor activity (6–8). In drug screenings, the antitumor agents are applied to the cells at the time of seeding and the cells are allowed to form colonies over 7-10 days (9). Therefore, it is also an important technique in estimating drug inhibitory effect (IC_50_) which is defined as the concentration of a drug required to inhibit the growth of colonies by half (8, 9). This dose-response curve allows us to determine the lowest concentration which has inhibitory effects and therefore less toxicity when administered in patients. Moreover, CFA is also used to measure biological damage to the cells after ionizing radiation treatment (10).

An arbitrary threshold is set for the definition of a colony (more than 15 cells). At the end point, the number of colonies is counted manually under a microscope, and parameters such as plating efficiency (PE) or survival fraction (SF) are evaluated to assess the efficiency of the assay (11). PE is calculated as the number of colonies formed at the final timepoint divided by the number of cells initially seeded, while SF is determined by dividing the number of colonies formed after treatment by the number of cells seeded and the PE.

Although a powerful tool to measure cell proliferative ability and to screen for the effectiveness of chemotherapy drugs, this method has many limitations. The availability of large amounts of samples is needed to seed 6-well plates and many plates to screen multiple drugs. Furthermore, it is extremely cumbersome to count the colonies under the microscope manually, and often individual biases make it complicated to obtain consistent results. Evaluating the colonies at the endpoint does not take into account the response of the cells to the drugs over time. Therefore, tracking not only colonies but also single cells and clusters over time provides a comprehensive understanding of proliferation rates of different cells, clonogenic potential, and resistance mechanisms (9, 11).

Automated CFA using high-throughput methods, such as conducting the assay in a 96-well microplate with fluorescence microscopy, presents a significant improvement over the traditional approach (12). The Agilent BioTek Cytation 5 cell imaging multi-mode reader, with its wide field of view, is particularly advantageous as it allows for efficient screening in a 96-well format, saving considerable time and resources. However, this method relies on staining cells with crystal violet or fluorescent dyes to visualize colonies, which introduces some limitations (13). Specifically, it does not allow for the tracking of live cells over time, thereby failing to capture the dynamic nature of colony formation. This lack of real-time monitoring could overlook crucial aspects of cellular behavior, particularly how colonies evolve and respond to treatments across different time points. Several AI-driven colony counting systems exist, such as Axion Biosystems’ Omni platform, which integrates live-cell analysis with an AI-powered clonogenic assay module. This system enables the automated evaluation of parameters such as PE and SF, reducing manual labor while increasing accuracy and reproducibility (7, 8, 11). However, current AI-based systems often have limitations, including high costs associated with proprietary microscopes and restricted plate formats, as is the case with Omni, which only supports 6-well plates. Additionally, many existing solutions are incompatible with pre-existing lab equipment and with microfluidic platform integration adding further cost. In a broader context, AI tools such as convolutional neural networks (CNNs) could be trained on colony images to improve colony detection and classification across various experimental conditions (14, 15). Moreover, open-source platforms like CellProfiler and ImageJ can be integrated with machine learning models for more flexible, cost-effective solutions. Additionally, transfer learning techniques, where pre-trained models can be fine-tuned to new datasets, also hold promise for improving colony recognition without requiring extensive computational resources or custom hardware (16–19). Although manual counting is still considered the gold standard, it is labor-intensive and can introduce variability and bias due to operator influence. To address these challenges, recent efforts have focused on automating cell identification and quantification (20). An automated colony counting software ideally should reduce the manual labor when it comes to colony counting, incorporate automated time-lapse microscopy to acquire as much information as possible to follow the formation of colonies over time and enable low-volume culture vessels to address low sample availability.

CFA is routinely used for drug screening and testing combination therapies in leukemia cells. In these assays, the leukemia cells are cultured in methylcellulose and exposed to increasing concentrations of various drugs. Colonies are counted 7-10 days after treatment to assess the effectiveness of the drugs (21, 22). Moreover, the colonies are counted at the endpoint which ignores the dynamics of colony formation over time. Acute Lymphoblastic Leukemia (ALL) is a hematologic malignancy of the lymphoid lineage that accounts for 25% of all childhood cancers. With multi-agent chemotherapy, the fatality of pediatric ALL has been reduced by ~90%. In adults, the response has not been as superior as in pediatric patients. Nevertheless, immunotherapies like CAR-T cell therapies, and CD-19 targeted T cell engagers have emerged as new treatment options specifically in B-cell ALL or B-ALL (23–25). ALL is classified based on the cell type (immature precursor of lymphoid lineage), immunophenotyping, and genetic features of the leukemic cells. When the malignancy arises from a precursor B-cell, it is termed a B-cell ALL and T-cell progenitor cells derived ALL are termed a T-cell ALL or T-ALL (26, 27). The E2A-PBX1 fusion gene plays a crucial role in the development of a specific subtype of B-ALL. This gene is formed by a translocation between parts of chromosomes 1 and 19 and is found in approximately 3-5% of pediatric B-ALL cases. In this study, we aimed to utilize the E2A-PBX1 pre-B-ALL cells from an established murine model to develop an Artificial Intelligence (AI)-assisted automated colony forming assay, integrating time-lapse microscopy and microfluidics-based drug screening to facilitate a time efficient method to accurately evaluate the effects of combination therapies. To ensure compatibility with microfluidic systems, we intend to use collagen as the matrix which can better mimic the natural *in-vivo* microenvironment (3, 22). Our main goal was to reduce labor-intensive manual counting and to get more accurate insights into the dynamics of colony formation throughout the experiment with the help of automated imaging and finally integrating a microfluidics-based approach to automate drug applications.

## 2 Materials and methods

### 2.1 Cell culture

The m159 primary cells used are derived from a population of B-ALL cells isolated from a mouse model expressing the E2A-PBX-1 fusion gene (22, 22). The E2A-PBX-1 positive mouse B-ALL cells, hereafter referred to as m159, were kindly provided by Prof. Dr. Jesús Duque-Afonso.

### 2.2 Reagents

For methylcellulose-based CFA, m159 cell suspension in complete IMDM [cIMDM, Iscove’s Modified Dulbecco’s Medium (Stem Cell) supplemented with 10% fetal bovine serum (FBS; Gibco), 1x L-glutamine (Gibco) and 1% Pen Strep (10,000 Units/ml Penicillin, 10,000 μg/ml Streptomycin; Gibco)] containing IL-7 (10 ng/ml, Stem Cell) was mixed with MethoCult™ (Stem Cell). For collagen-based CFA, m159 cell suspension in cIMDM containing IL-7 was mixed with a collagen solution (1.09 mg/ml). This collagen solution was prepared using Collagen Type I Rat Tail (5 mg/ml, Ibidi) and 1 M NaCl, 7,5% NaHCO_3,_ and millipore H_2_O. For IC_50_ determination, JQ1 (S7110, Selleckchem), Prednisolone (PRDL, S1737, Selleckchem), and Daunorubicin (DNR, S3035, Selleckchem) were tested.

### 2.3 Transitional methodology from traditional to automated collagen-based CFA

A density of 50,000 cells/ml of m159 cells were cultured in both collagen-based and methylcellulose-based media. 60 µl of each cell suspension was seeded in triplicate in a µ-Slide 15-well 3D (Ibidi) and incubated in the BioTek Lionheart FX Automated Microscope. A stage-top incubator system (Ibidi) was coupled to the microscope to maintain controlled conditions of temperature (37°C), CO_2_ (5%), and humidity (95%). Time-lapse images were captured every 12 h for 4 days. Six regions of interest (ROIs) were analyzed per well, and brightfield images were acquired at 10x magnification. The absolute cell number was determined by manually counting single cells, clusters, and colonies at both the start and end time points. Colony-forming efficiency (% PE) was calculated as the number of colonies formed at the end time point divided by the number of single cells at the start point.

### 2.4 Collagen-based CFA for cell density optimization

Different cell densities of m159 cells (25,000, 50,000, 100,000, and 150,000 cells/ml) were cultured in a collagen-based medium. In parallel, 25,000 cells/ml were cultured in a methylcellulose-based medium as a positive control. Each cell density was seeded in triplicates at 60 µl per well in a 15-well slide and incubated in the UC2 Investigator Automated Microscope (UC2i) at 37°C, 5% CO_2,_ and 95% humidity controlled by the stage top incubator system. The UC2i microscope was developed in-house in cooperation with LABMaiTE GmbH & OpenUC2 GmbH. Image acquisition was performed every 8 h for 5 days and consisted of 25 Z-slices with 30 µm separation, resulting in a total height of 750 µm. Two ROIs were considered per well and brightfield images were captured at 10x magnification. Z-stacks of time-lapse images generated were annotated using Roboflow (28), an annotation tool for ground truth labeling, with the labels single-cell, cluster, and, compact- and dispersed-colony. Additionally, any out-of-focus clump of cells that could become a cluster or colony was labeled as cluster-candidate or colony-candidate, respectively (**Supplementary Figure S1**). Once annotated, the dataset was exported and used for AI model training.

### 2.5 Automated colony counting using deep learning & multi-object tracking

The analysis process for counting colony formation on microscopy Z-stacks consists of two major stages. The first stage is the localization and classification of objects on a single 2D image or slice of the Z-stack which results in a list of objects as well as their class, coordinates, and size of the enclosing rectangle around the object, per image. We apply the multi-object tracking (MOT) BOTSort algorithm to merge the slice information along the Z-stack to form a 2.5D representation of unique objects and their location (29).

The approach was chosen as colony formation in a 3D culture usually involves scanning along a large vertical range to capture all colonies growing in different layers of the material. This eliminates the possibility of merging objects strictly by their x and y location as this could combine multiple colonies into a single detection. Multi-object tracking is usually applied in the temporal dimension where the spatial movement of objects is tracked and objects are likely to disappear or be occluded occasionally and therefore have to be re-identified but not confused with other objects traveling across the same location in the meantime. This domain can be easily reformulated to fit the need for tracking objects along a Z-stack as similar events such as positional drift, out-of-focus, or occlusion by another object provide an equal challenge here.

Given the substantial volume of data generated per imaged position and the large number of unique positions captured in each experiment, we decided on a real-time object detection model, specifically the YOLOv8-m model provided by the *ultralytics* library (30). YOLOv8 is a popular, efficient, and powerful Deep Convolutional Neural Network that can perform real-time bounding box detection and instance segmentation tasks. We attempt to keep the computational cost of the analysis process low to keep this solution accessible for consumer-level hardware and preserve reasonable analysis runtime for a full experimental analysis of the 11520 image slices per chip in these experiments. Real-time models usually sacrifice accuracy for faster inference speed which is why we expect a lower performance compared to larger but slower models. This work however investigates the feasibility of the method in general which can be easily adapted to incorporate a different model once its applicability has been shown.

Individual object detections on each independent slice of a stack are combined into trajectories using the BOTSort tracking algorithm. Classification of a complete track is done by taking the maximum of all classes present in the trajectory as class IDs are ordered hierarchical in terms of importance or relevance, i.e. candidates (not relevant unless actual class detected), cells (a subset of following classes), cluster (consisting of cells, a subset of a colony), colony (extended definition of a cluster). A typical trajectory starts with an object being recognized as a candidate first since by definition these are objects that are out of focus (and therefore not reliably classifiable) but their shape and size allow the hypothesis that a larger, relevant object will come into focus. It is followed by one or multiple cluster or colony detections after again moving out of focus and becoming a candidate again. This approach provides reliable classification as multiple focal planes are considered in the classification procedure as long as the true class is at least detected in one slice of the stack.

The detector model was trained on a dataset of 82 microscopy images (4024x3036 pixels) containing 2.732 annotated objects across 4 distinct classes: candidate objects, individual cells, cell clusters, and colonies. Due to ambiguity and under-representation of the cluster-candidate, colony-candidate as well as dispersed- and compact colony classes in the original dataset, we decided to combine these into the *candidate* and *colony* classes respectively. Each training image was augmented to produce 2 additional images to enhance the robustness and generalization of the model. The choice of augmentation transformations included 90-degree rotations, random cropping (0-25% zoom), random rotations (-45° to +45°), brightness adjustments (± 15%), exposure modifications (± 5%), and Gaussian blur up to 3.6 pixels, resulting in 246 training images. In addition, 29 images have been held out from the training data and augmentation and are used to evaluate the detector performance after the hyperparameters for the detection and tracking have been jointly optimized using the Optuna framework (31). We optimize the parameters at the same time as the tracking behavior heavily depends on what kind of detections are generated by the upstream model. Note that the used hyperparameters of the detector (confidence, NMS threshold, and runtime augmentations) only alter the inference behavior but the underlying trained model will remain the same.

Separately, five Z-stacks (19 slices per stack, time points 8 and 10) taken from existing experiments have been annotated using CVAT with MOT annotations to perform and evaluate the tracking procedure (32). Each track was assigned the proper class using the maximum operator according to the previous definition. To reduce the annotation effort, only cluster and colony classes have been annotated as these are the primary classes considered in this work and following experiments. The python library *pymotmetrics* was used to evaluate the tracking output and compute the necessary MOT scores (33). Optuna was used to jointly optimize the YOLOv8 detection and BOTSort hyperparameters for 100 iterations on one of the five annotated Z-stacks. The product of the IDF1 score and percentage of objects tracked (mostly-tracked and partially tracked, relative to total object count) was used as the optimization objective. We aim to maximize this objective and include the tracked percentage metric to emphasize recall of the model over precision as consistent annotation of in-focus vs. out-of-focus in this setting turned out to be very challenging. Hyperparameters and details for the training of the YOLOv8 model can be found in **Supplementary Figure S2**. The final optimized parameters for both YOLOv8 inference and BOTSort which were used for the analysis of the biological results can be found in **Supplementary Figure S3**.

### 2.6 Collagen-based CFA for IC_50_ determination

A 5-point drug titration was set up as follows. For JQ1, PRDL, and DNR, a 10x dilution was prepared from the stock in cIMDM, followed by 1:5 serial dilutions. The tested concentrations were 500 nM, 100 nM, 20 nM, 4 nM, and 0.8 nM for JQ1, 125 nM, 25 nM, 5 nM, 1 nM, and 0.2 nM for PRDL, and 25 nM, 5 nM, 1 nM, 0.2 nM, and 0.04 nM for DNR. m159 cells (100.000 cells/ml) were resuspended in Megacult™(Stem cell)with IL-7 and then mixed with collagen solution and the corresponding 10x dilution of each drug to achieve a 1x final concentration. Controls include m159 cells mixed with dimethyl sulfoxide (DMSO, 1:1000, Sigma) as negative control and with cIMDM as an untreated (UT) control. JQ1, PRDL, DNR, and control groups were all tested in the same experiment using a 4-slide adapter (Ibidi) coupled to the UC2i microscope. Each slide contained triplicates of each dilution/condition per drug. Image acquisition was performed every 12 h for 5 days and consisted of 20 Z-slices with 30 µm separation, resulting in a total height of 600 µm. Two ROIs were considered per well and brightfield images were captured at 10x magnification. Data were normalized to the DMSO control. The IC50 was calculated by fitting a sigmoidal dose-response curve to the normalized total number of colonies and clusters.

### 2.7 Statistical analysis

**Figure 1:**
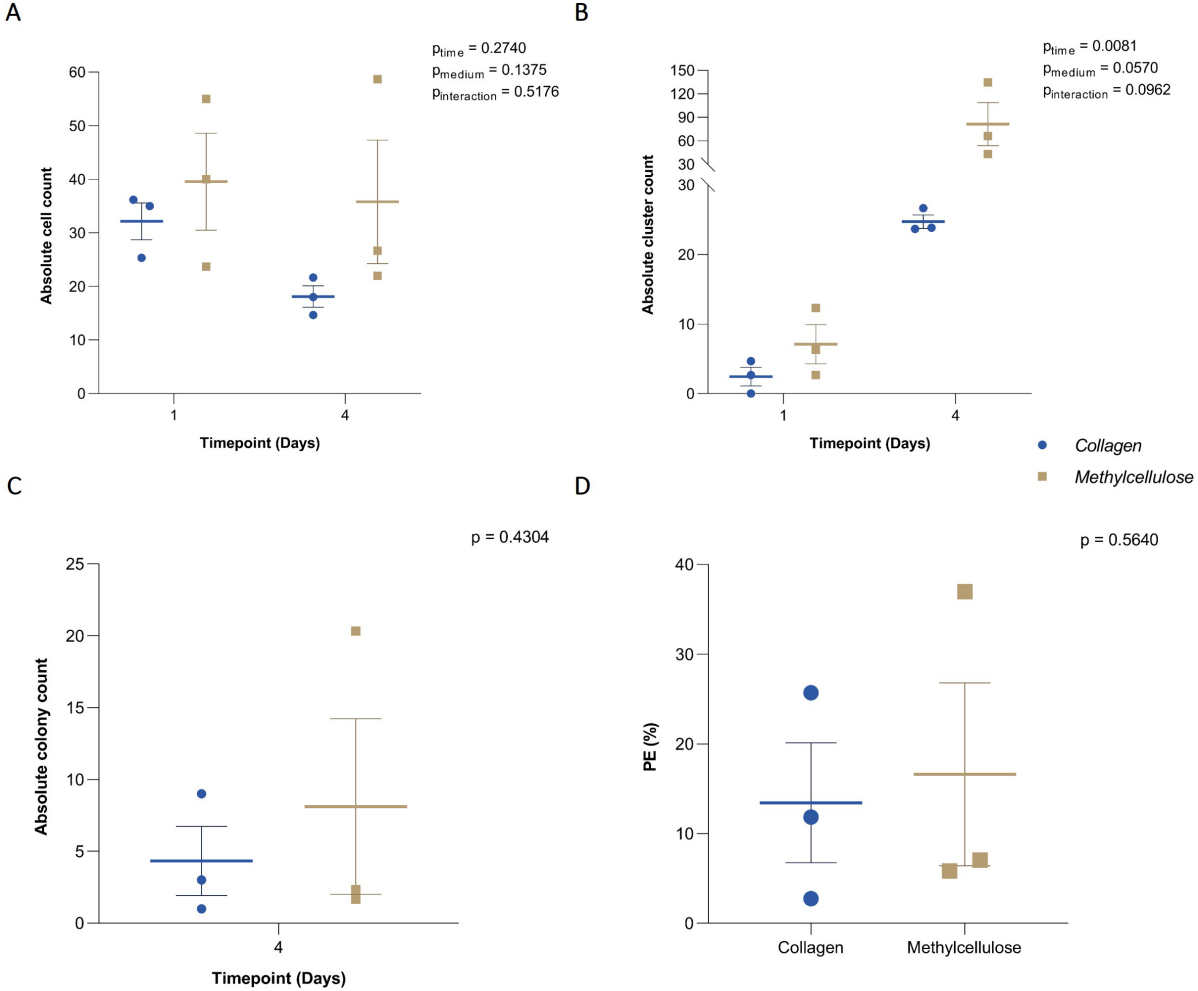
Efficiency of collagen-based CFA. **(A)** Absolute cell count, **(B)** clusters, and **(C)** colonies grown on collagen- and methylcellulose-based media and manually counted at baseline and after 4 days of incubation. **(D)** Plating efficiency (PE) in collagen and methylcellulose-based media after 4-day incubation. Individual results from each independent experiment (n = 3) are plotted alongside the mean ± SEM.

**Figure 2:**
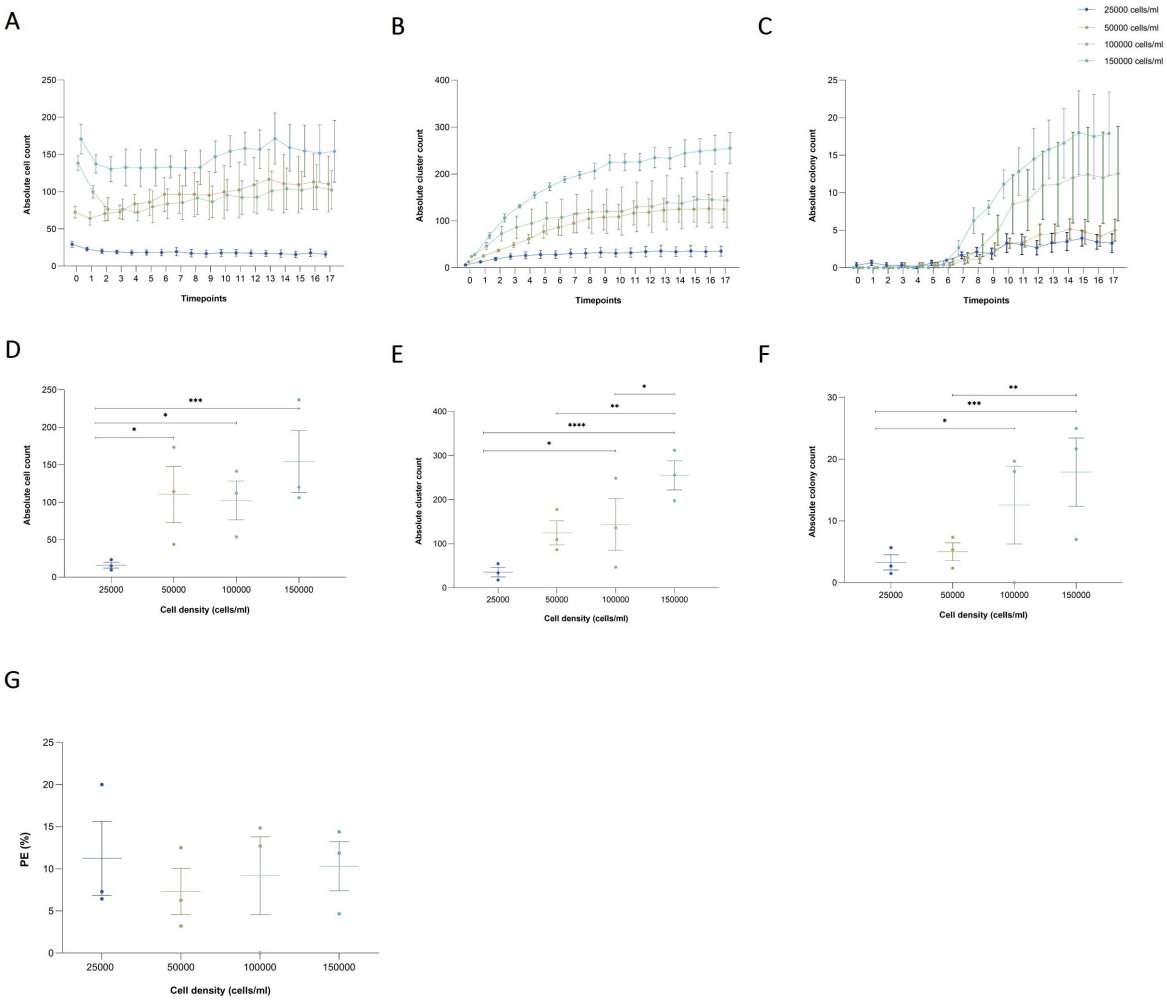
Effects of seeding cell density on cell, cluster, and colony counts in a collagen-based CFA. **(A)** Absolute cell count, **(B)** cluster, and **(C)** colony over time for varying initial seeding densities (25,000, 50,000, 100,000, and 150,000 cells/ml) enumerated by the AI-detection model. Results are presented as the mean ± SEM from three independent experiments. **(D)** Absolute counts of single cells, **(E)** clusters, and **(F)** colonies at the final time point (day 4) across the different cell densities. Individual results from independent experiments are presented, along with mean ± SEM. Statistically significant differences between groups are indicated by asterisks (*p < 0.05, **p < 0.01, ***p < 0.001, ****p < 0.0001). **(G)** PE across different seeding cell densities after 4 days of incubation. Individual results from independent experiments are presented, along with mean ± SEM.

**Figure 3:**
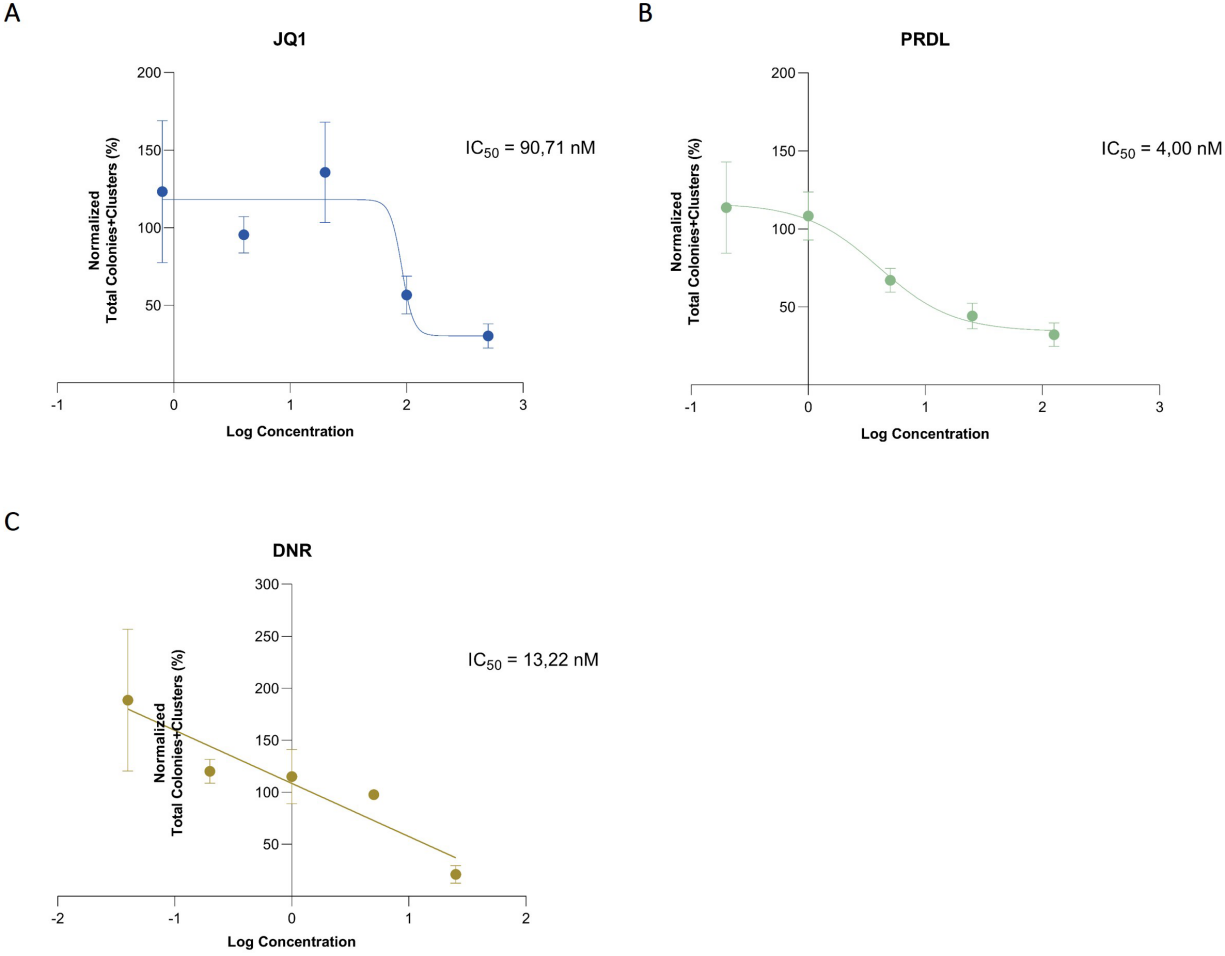
IC_50_ determination using data generated by the AI software. Sigmoidal curves using a 4-parameter nonlinear model for **(A)** JQ1 and **(B)** PRDL and linear regression for **(C)** The total number of colonies and clusters was normalized to the DMSO control and the concentrations were represented in logarithmic scale. Results are presented as the mean ± SEM from three independent experiments.

Raw data from the AI-detection model was further processed and statistical analyses were performed using GraphPad Prism 8 software (version 8.4.3). Each experiment was performed in triplicate. For the comparison between methylcellulose and collagen-based media, data of cell, and cluster counts were analyzed using a two-way ANOVA Results 3.1, **Figures 1A–C**, while a Student’s t-test was used to analyze colony counts and PE data **Figure 1C**. In the cell density experiments shown in Results 3.3, **Figures 2A–F**, comparisons were made using a two-way ANOVA to assess the effects of seeding cell density and time, while one-way ANOVA was used to analyze differences in PE **Figure 2G**. P values < 0.05 were considered significant. For the IC_50_ determination experiments (Results 3.4, **Figure 3**), the dose-response curve was made by fitting a nonlinear curve (sigmoidal, 4-parameter model) of the normalized colony and cluster count on a logarithmic scale for JQ1 and PRDL, and a simple linear regression curve of the normalized colony and cluster count on a logarithmic scale was used to estimate IC_50_ for DNR. Results are displayed as each individual experiment or as the mean of the three experiments with the corresponding standard error of the mean (SEM).

## 3 Results

### 3.1 Establishing a collagen-based CFA

To develop a robust, user-friendly, and microfluidics-compatible CFA, the traditional methodology was modified by specifically replacing the methylcellulose medium with collagen for cell growth and significantly reducing the volume used. The m159 cells were cultured in collagen-based media supplemented with Megacult and IL-7, seeded in a 15-well slide, and incubated under controlled conditions in the microscope at 37°C with 5% CO_2_ and 95% humidity. Images were acquired every 12 h, consisting of 10 Z-slices with 10 µm separation, covering a total height of 100 µm. Single cells, clusters, and colonies within 6 ROIs were manually counted at the start and after a 4-day incubation. Only in-focus Z-stacks were included to avoid overcounting. While images were taken at 12-h intervals, data analysis was focused on the initial and final time points.

To assess the efficiency of colony formation in a collagen-based medium in a low-volume culture vessel and to confirm its comparability to a methylcellulose-based medium, the absolute cell counts and their capability to proliferate in both media were evaluated. For single cells, no significant effect of the matrix, time, or interaction between these factors was detected (p_matrix_ = 0.1375, p_time_ = 0.2740, and p_interaction_ = 0.5176). Accordingly, no differences were observed in the total number of single cells grown in collagen compared to methylcellulose medium at any time point **Figure 1A**. Clusters, defined as groups of 2-14 cells, showed a significant effect of time (p_time_ = 0.0081), but neither the matrix nor interaction had a significant effect (p_matrix_ = 0.0570, and p_interaction_ = 0.0962) **Figure 1B**. Colonies, defined as groups of more than 15 cells, were only analyzed at the final time point since only single cells or small clusters were present initially. No significant differences were found in the number of colonies formed in the collagen-based medium compared to the methylcellulose-based medium (p = 0.4304) **Figure 1C**. Lastly, the colony-forming efficiency (PE) after 4 days was similar in both matrices (p = 0.5640) **Figure 1D**. These results demonstrate the efficiency of scaling down from the traditionally used CFA volume of 3 ml down to the 60 µl volume used in these experiments. Indeed, the ability of B-ALL cells to grow and form colonies in a collagen matrix was not affected, making it an excellent option for use in combination with microfluidics.

### 3.2 Automated colony counting using deep learning & multi-object tracking

We evaluated both, colonies and clusters together as well as colony and cluster tracking individually to assess the class-based performance (only ground truth and detections for specific class considered). We averaged the results per evaluation across the four tracking datasets which have an average unique object count of 96.5 clusters and 28 colonies. Averaged and individual dataset results can be seen in **Table 1**. Note that the dataset with id 126 is not present as this dataset was used for the hyperparameter optimization. We focused on the IDF1 score, which is the F1-Score adapted to the MOT scenario, as well as the mostly- and partially-tracked metric, which measures how many of the unique objects have been detected by the pipeline. Similarly, the mostly-lost metric reflects how many objects have not been tracked or less than 20% of their trajectory.

**Table 1:** Detection & tracking results.

Dataset	Class	# Objects	Mostly tracked	Partially tracked	Mostly lost	idf1	idp	idr
Average	all	124	76	35	13.25	0.752	0.739	0.770
Average	cluster	97	61	23	13.00	0.728	0.692	0.768
Average	colony	28	15	12	1.50	0.805	0.850	0.772
123	cluster	91	49	24	18	0.682	0.670	0.695
123	colony	28	15	12	1	0.831	0.927	0.752
123	all	119	64	36	19	0.730	0.746	0.715
124	cluster	103	78	14	11	0.764	0.696	0.847
124	colony	14	14	0	0	0.887	0.839	0.940
124	all	117	93	14	10	0.788	0.719	0.872
125	cluster	80	51	17	12	0.749	0.713	0.789
125	colony	31	10	17	4	0.704	0.800	0.629
125	all	111	62	36	13	0.744	0.751	0.738
127	cluster	112	64	37	11	0.715	0.690	0.739
127	colony	39	21	17	1	0.799	0.835	0.766
127	all	150	85	54	11	0.748	0.739	0.753

MOT scores of the analysis pipeline were evaluated on four MOT datasets after being optimized on a separate dataset using Optuna.

# Objects represent the number of unique objects and their trajectories present in the dataset. The “Average” dataset represents the averaged scores across the four datasets. Mostly and Partially tracked are considered a successful recognition while Mostly lost indicates untracked objects.

**Figure 4:**
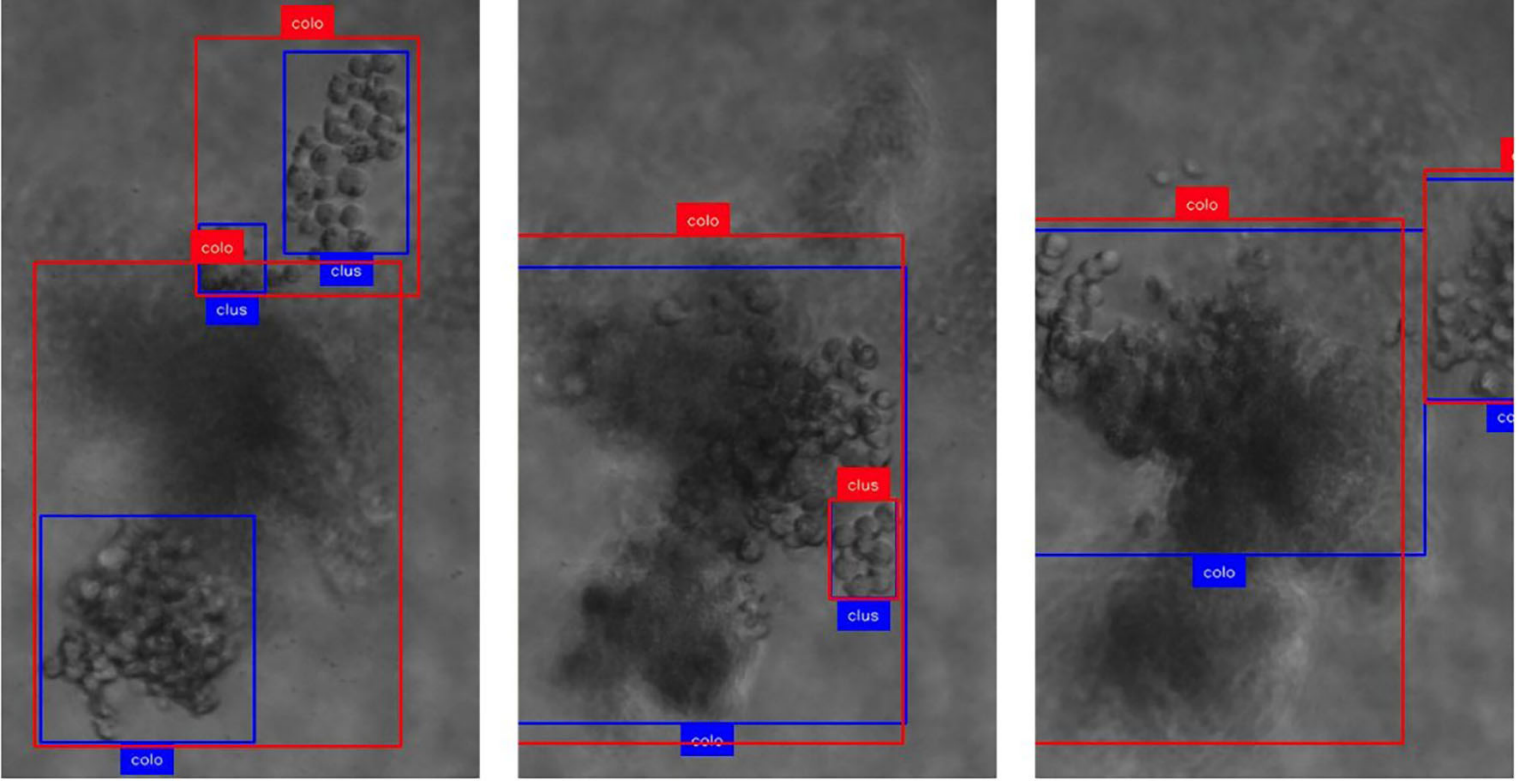
Demonstration of focus-related challenges for the detection and tracking mechanism. Boxes in red show ground truth annotations, and blue display model predictions. The text represents the class provided by ground truth and model, respectively. (Col, colony; clus, cluster). As the focal plane progresses along z (left to right), specific parts of the colony are detected and tracked which leads to rapid changes in the (center-) location of the object.

We found an IDF1 score of 0.752 with 61.2% of objects tracked more than 80% of their trajectory (mostly-tracked), 28.2% between 80% and 20% (partially-tracked) and 10.6% failed to be recognized by our system (mostly-lost). The majority of the missed objects appeared to be smaller clusters caused by inconsistencies in the annotations. Out of the total of 112 colonies present in the dataset, only 3 are missed by the pipeline (2.7%). These misses can be explained by large colonies that span across a wide range of focal planes, exposing different portions of the colony in focus at each step and therefore the detection model recognizing only portions of the colony in contrast to the complete colony including the out-of-focus parts (**Figure 4**). These large changes in coordinates cause the tracker to not be able to associate the objects, therefore failing to combine them into a single trajectory. Similarly, the partial detection of colonies can also lead to a recognition of a large colony as multiple smaller sub-colonies, resulting in an overestimation of colony count. Both issues should be addressable by revisiting the annotation criteria of colonies and including out-of-focus parts to get more consistent large colony detection. Furthermore, we observed that the general definition of cluster (15 cells or less) vs. colony (more than 15 cells) generally seems to be picked up well by the model but occasionally sees misclassifications towards the decision boundary.

**Figure 5:**
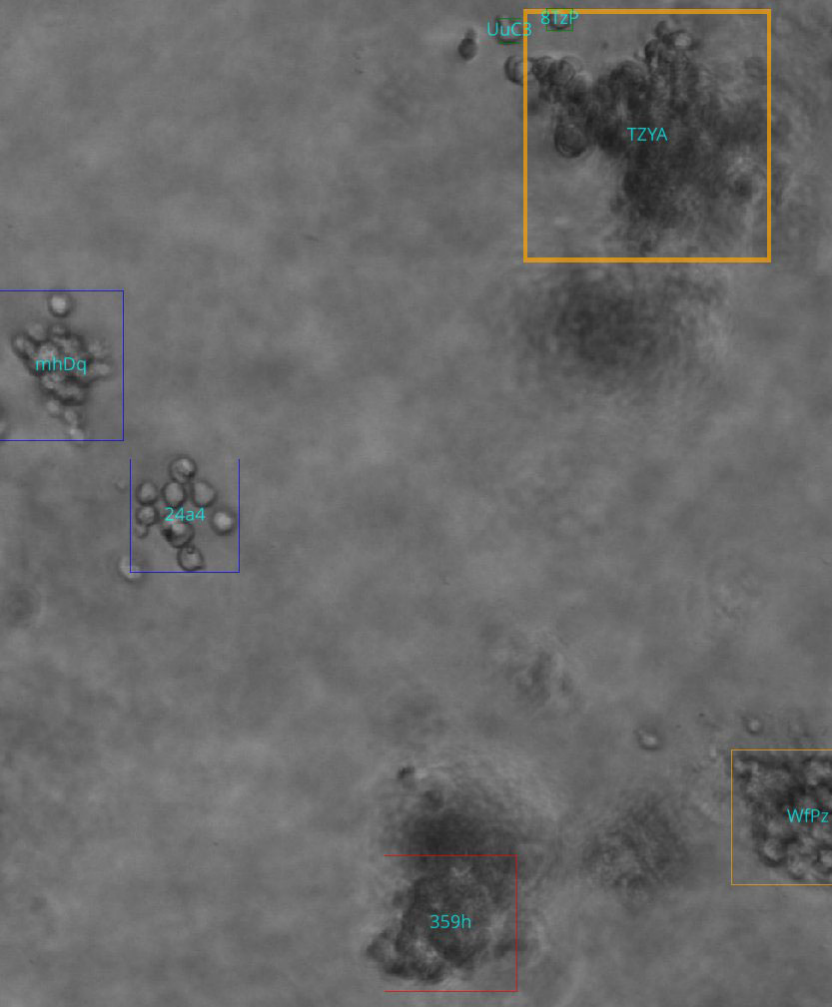
Example output of the analysis pipeline. Box colors represent classification output at the current z-index, and text inside the box represents the unique track ID assigned. Colors represent class (red: candidate, green: single cell, blue: cluster, yellow: colony).

We also noted that our experiment shows no ID-switching errors, indicating that the tracker is capable of re-associating objects very reliably which is important as ID switches would cause a single true object to be counted multiple times. Overall, a qualitative inspection yielded a reliable tracking and detection mechanism with occasional errors in edge cases. An example of the pipeline output can be found in **Figure 5**.

With optimized parameters for the detector and the tracking algorithm, we can additionally evaluate the detector on the initial manual annotations to assess the frame-by-frame classification performance. In contrast to the tracking datasets, annotations for candidates and single-cell classes are also present and the classification performance for these objects can also be assessed.

**Figure 6:**
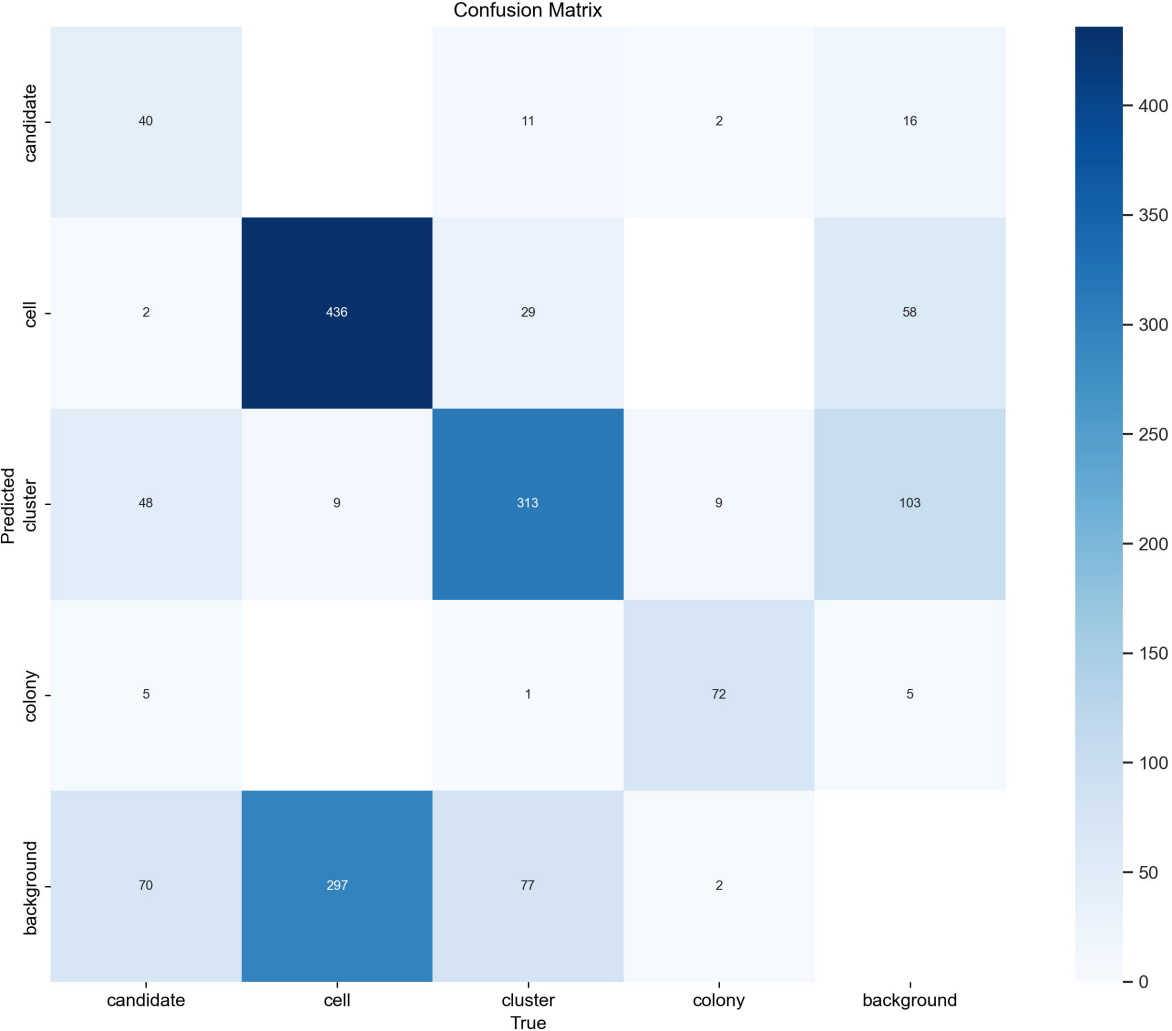
YOLOv8 confusion matrix. The confusion matrix of the final object detector was evaluated on 29 test images after hyperparameter optimization was performed on the tracking datasets. Rows represent class predictions while columns represent true ground truth annotations. Diagonal elements represent correct predictions while off-diagonal elements represent misclassifications. The number in each entry corresponds to the number of times this combination of prediction vs. ground truth occurred.

We found that the evaluation results on the 29 ground truth images confirm the results seen in the tracking evaluation. The detector achieved a mean Average Precision (mAP) of 0.668 at an Intersection over Union (IoU) threshold of 50% across all classes. **Table 2** shows that performance varied considerably between classes, with colonies showing the highest mAP50 of 0.861, followed by clusters (0.722), cells (0.697), and candidates (0.392). The lower scores for clusters and cells can most likely be explained by inconsistencies in the ground truth due to the different focal planes and subjective decisions on which potential object to annotate and which one is too far out of focus. The confusion matrix shown in **Figure 6** supports this by revealing a large number of supposedly false positives for single cells and clusters, which upon inspection turn out to be unlabeled objects, either due to the focus conditions or due to the object being enclosed by a larger object (e.g. cells within a cluster annotation). Only in rare cases actual false positives, e.g. detecting debris are seen which can be explained by limited examples of debris in the training dataset. Overall, the results show that the detection model can correctly recognize the majority of objects in the dataset although with confusion in the classification caused by overlapping or inconsistent class definitions.

**Table 2:** Object detection evaluation results.

Class	Images	Instances	Precision	Recall	mAP50	mAP50-95
all	29	1423	0.722	0.594	0.668	0.451
candidate	18	165	0.565	0.236	0.392	0.27
cell	27	742	0.819	0.58	0.697	0.356
cluster	28	431	0.647	0.724	0.722	0.492
colony	22	85	0.855	0.835	0.861	0.685

Evaluation results of the YOLOv8 object detector on the 29 manually annotated test images, separated by class. mAP is the mean Average Precision and represents the average precision between ground truth and prediction boxes at different overlap thresholds (50%, 50-95%).

### 3.3 AI-assisted CFA for cell density optimization

After establishing the collagen-based CFA, we aimed to utilize this setup to optimize and select the most suitable cell seeding density that leads to the most optimal PE, ensuring consistent and reliable colony formation results. Furthermore, the initial seeding density mustn’t lead to overcrowded colonies which makes it difficult to track and count manually as well as for the AI detection model.

The detection model recognized, counted, and tracked single cells, clusters, and colonies throughout the time. For single cells, the absolute count remained consistent over time across all tested seeding densities. A significant effect of cell density on cell number was observed, but there was no effect of time or the interaction between both factors (p_cell density_ < 0.0001, p_time_ = 0.8253, and p_interaction_ > 0.9999) **Figure 2A**. By the final time point, after 8 days of incubation, the number of single cells in the lowest density group (25,000 cells/ml) was significantly lower than in the 50,000, 100,000, and 150,000 cells/ml groups (p = 0.0181, p = 0.0366, and p < 0.0001, respectively) **Figure 2D**. For clusters, an increase in the absolute count was observed starting at 12 h of incubation, after which the number remained constant until the last time point in all evaluated groups. A significant effect of both cell density and time on the number of clusters was detected, but not of their interaction (p_cell density_ < 0.0001, p_time_ < 0.0001, and p_interaction_ > 0.7832) **Figure 2B**. At the final timepoint, although the total number of clusters formed in the 25,000 cells/ml group was lower than in the other groups, it was only significantly different from the 100,000 and 150,000 cells/ml groups (p = 0.0237, and p < 0.0001, respectively). While the total number of clusters in the 150,000 cells/ml group was higher than in the other groups, it was only significantly different from the 50,000 and 100,000 cells/ml groups (p = 0.0039, and p = 0.0191, respectively) **Figure 2E**. For colonies, an increase in the absolute count was observed after 3 days of incubation across all evaluated groups. A significant effect of both cell density and time on colony number was detected, though there was no significant interaction between these factors (p_cell density_ < 0.0001, p_time_ < 0.0001, and p_interaction_ = 0.0547) **Figure 2C**. At the final time point, although the mean number of colonies in the 25,000 cells/ml group was lower than in the other groups, it was only significantly different from the 100,000 and 150,000 cells/ml groups (p = 0.0419, and p = 0.0003, respectively). Similarly, while the mean number of colonies in the 150,000 cells/ml group was higher than in the other groups, it was only significantly different from the 50,000 cells/ml group (p = 0.0017) **Figure 2F**. Finally, no significant differences were observed in PE among the groups tested (p = 0.8931) **Figure 2G**. As a result, the key criterion for selecting the optimal seeding density was based on colony visualization and ensuring adequate space for cells to grow. Based on the findings, we determined that 100,000 cells per well is the optimal seeding density for future experiments. Manual colony counting would yield only a final count for each density, making the process both time-consuming and labor-intensive. The implementation of AI-assisted CFA reduced the time and effort required to analyze a large number of samples, while also providing deeper insights into colony formation dynamics, including single-cell and cluster counts over time.

### 3.4 AI-assisted CFA for IC_50_ determination

**Figure 7:**
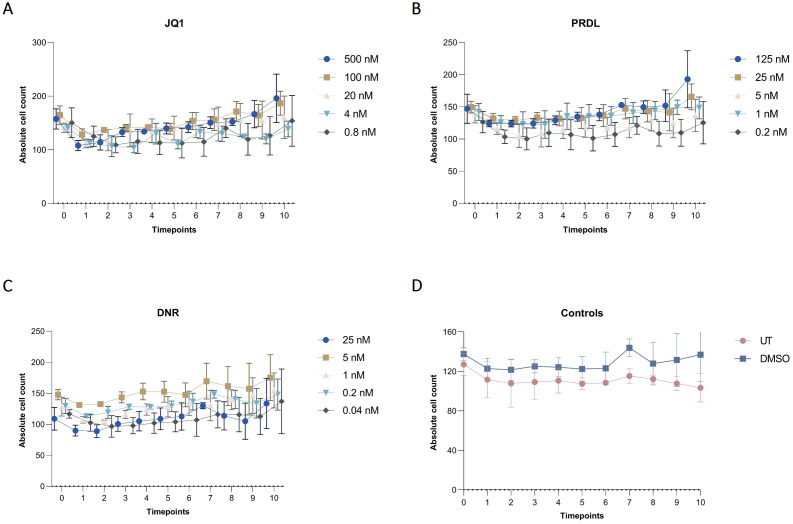
Cell dynamics for different drugs and concentrations in collagen-based CFA over time. Absolute cell counts for m159 cells treated with different concentrations of **(A)** JQ1 (500 nM, 100 nM, 20 nM, 4 nM, and 0.8 nM), **(B)** PRDL (125 nM, 25 nM, 5 nM, 1 nM, and 0.2 nM), **(C)** DNR (25 nM, 5 nM, 1 nM, 0.2 nM, and 0.04 nM), and **(D)** control groups, including untreated (UT) and DMSO-treated cells enumerated by the AI-software. Results are presented as the mean ± SEM from three independent experiments.

**Figure 8:**
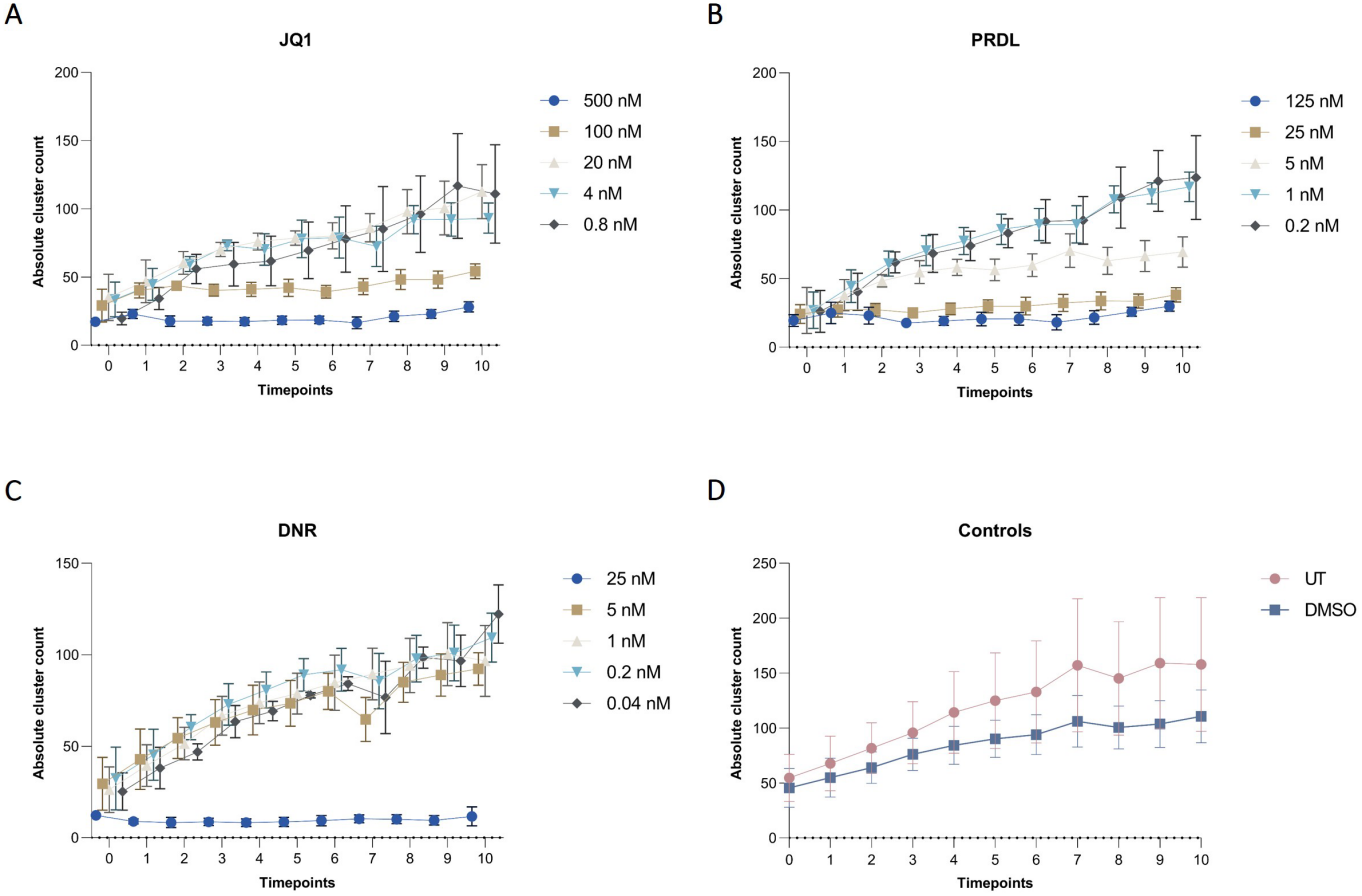
Cluster dynamics for different drugs and concentrations in collagen-based CFA over time. Absolute cluster counts for m159 cells treated with different concentrations of **(A)** JQ1 (500 nM, 100 nM, 20 nM, 4 nM, and 0.8 nM), **(B)** PRDL (125 nM, 25 nM, 5 nM, 1 nM, and 0.2 nM), **(C)** DNR (25 nM, 5 nM, 1 nM, 0.2 nM, and 0.04 nM), and **(D)** control groups, including untreated (UT) and DMSO-treated cells enumerated by the AI-software. Results are presented as the mean ± SEM from three independent experiments.

**Figure 9:**
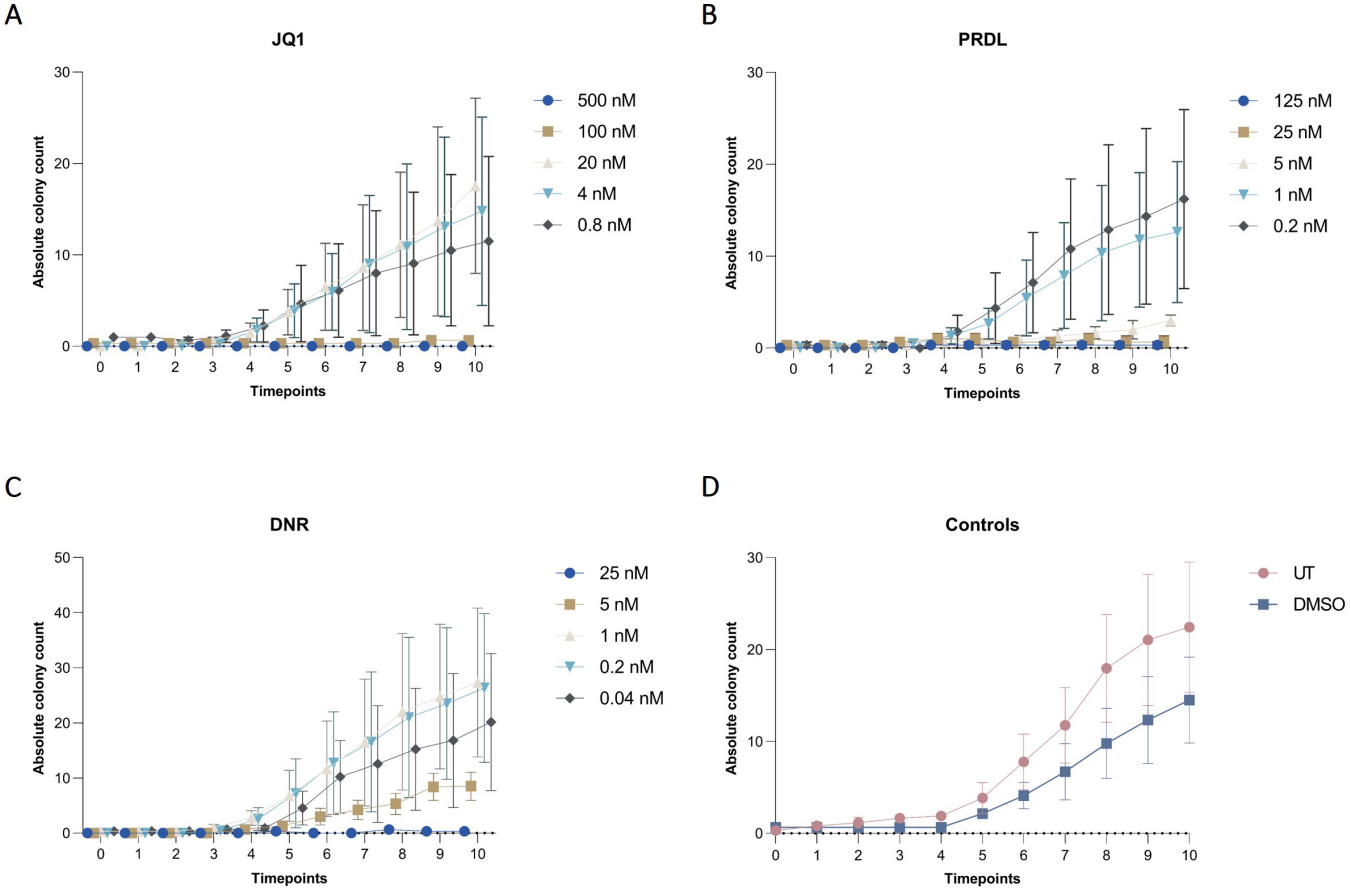
Colony dynamics for different drugs and concentrations in collagen-based CFA over time. Absolute colony counts for m159 cells treated with different concentrations of **(A)** JQ1 (500 nM, 100 nM, 20 nM, 4 nM, and 0.8 nM), **(B)** PRDL (125 nM, 25 nM, 5 nM, 1 nM, and 0.2 nM), **(C)** DNR (25 nM, 5 nM, 1 nM, 0.2 nM, and 0.04 nM), and **(D)** control groups, including untreated (UT) and DMSO-treated cells enumerated by the AI-software. Results are presented as the mean ± SEM from three independent experiments.

The applicability of the AI-assisted CFA developed was further evaluated by assessing the ability of B-ALL cells to form colonies and clusters after treatment and determining the IC_50_ for different drugs. Furthermore, the effects of the individual drugs on the number of cells, clusters, and colonies over time provide insights into the effectiveness of the treatments. To begin, IC_50_ values for JQ1, DNR, and PRDL were established using the traditional CFA with manual colony counting as a reference (data not shown). Based on these values, five concentrations within the upper and lower IC_50_ range were selected to evaluate the performance of the detection model to detect and track single cells, clusters, and colonies over time. The AI software accurately detected the three categories generating raw data that included the absolute number of single cells, clusters, and colonies for each ROI, time point, and Z-plane across all wells. Consequently, single-cell, cluster, and colony counts were analyzed over time for all tested drug concentrations and control groups. The absolute cell count followed a consistent pattern, remaining stable over time across all evaluated groups, including those treated with JQ1, PRDL, and DNR, as well as the control groups (DMSO and UT) (**Figure 7**). Regarding cluster formation, the absolute cluster count remained constant over time in cells treated with higher concentrations of JQ1 (500 nM and 100 nM), PRDL (125 nM, 25 nM, and 5 nM), and DNR (25 nM), whereas it increased over time in cells treated with lower concentrations of JQ1 (20 nM, 4 nM, and 0.8 nM), PRDL (1 nM and 0.2 nM), and DNR (5 nM, 1 nM, 0.2 nM, and 0.04 nM) (**Figures 8A–C**, respectively). In the control groups, both UT and DMSO-treated cells showed a steady increase in the absolute cluster count, following a similar pattern **Figure 8D**. In terms of colony formation, it was completely inhibited in cells treated with higher concentrations of JQ1 (500 nM and 100 nM), PRDL (125 nM, 25 nM, and 5 nM), and DNR (25 nM), as the absolute colony count remained at zero over time. In contrast, the absolute colony count increased after approximately 60 h of incubation in cells treated with lower concentrations of JQ1 (20 nM, 4 nM, and 0.8 nM), PRDL (1 nM and 0.2 nM), and DNR (5 nM, 1 nM, 0.2 nM, and 0.04 nM) (**Figures 9A–C**, respectively). Similarly, for the control groups (both UT and DMSO-treated cells), colonies began to form after approximately 60 h of incubation, with the absolute colony count steadily increasing over time **Figure 9D**.

For the determination of IC_50_, absolute counts for each ROI and Z-plane at the final time point across all conditions were estimated using the presented AI-detection model. In this context, the total number of colonies and clusters was considered to optimize the model’s fit and accurately estimate the IC_50_, thereby reducing the standard deviation and improving the r^2^ value. The estimated IC_50_ values were 90.71 nM for JQ1, 4 nM for PRDL, and for DNR 13,22 nM (**Figure 3**).

## 4 Discussion

Transitioning from a traditional CFA approach to an AI-assisted automated assay offers numerous advantages, including the reduction of labor-intensive manual counting tasks, elimination of human error, increased throughput, real-time monitoring, and automated analysis. CFA has long been a foundational technique for evaluating the effects of various drugs in ALL. In this study, together with our collaborators, we aimed to transform the traditional approach of CFA for a certain type of murine E2A-PBX1 B- ALL cells to a more efficient AI-assisted CFA approach coupled with time-lapse microscopy.

Our first approach was to scale down from a 6-well plate format to a 15-well slide format, thereby enhancing throughput even with limited sample quantities. This miniaturization, reducing the traditional 3 ml requirement in CFA to just 60 µl, not only preserves the ability of B-ALL cells to grow and form colonies effectively but also offers a major economic advantage. While not the primary focus of this study, the reduced volume requirement could greatly benefit research groups enabling them to conduct many more tests with the same amount of resources. Moreover, the small volumes required make it particularly advantageous when working with limited patient samples, maximizing the use of precious biological material. This scalability and compatibility with microfluidic platforms make this method an attractive option for wider adoption in various experimental setups.

Secondly, we aimed to integrate microfluidics-based approaches for automated drug application. Therefore, we focused on finding an alternative to methylcellulose as a semi-solid medium as we found it to be unsuitable for perfusion-based assays. We tested and found rat tail collagen to be a good alternative for methylcellulose as it did not dissolve during perfusion-based assays. Subsequently, we validated that the scaling down approach and switching to collagen did not show any significant differences in colony formation. The strategy behind developing an automated CFA involved leveraging automated time-lapse microscopy to continuously record cell colony formation over time. These images were then used to train an AI detection model capable of accurately identifying single cells, clusters, and colonies, and providing comprehensive analysis of critical parameters such as PE, which is essential for effective drug screening. Furthermore, tracking single-cell and cluster counts at multiple time points, rather than relying solely on endpoint colony counts as in traditional CFAs, provides a deeper understanding of cell behavior dynamics, such as differential proliferation rates and resistance mechanisms. Investigating single cells that persist after drug exposure and evade apoptosis could also offer critical insights for evaluating the efficacy of different therapeutic agents. Given that time-lapse microscopy enables prolonged observation of cells, it naturally generates an enormous amount of data. To manage this, we strategically limited imaging to 2 to 4 ROIs within each well. Additionally, because the cells are embedded in collagen and grow in a three-dimensional manner, Z-stacks were employed to capture the Z-plane, ensuring that the full depth and structure of the colonies were accurately recorded and analyzed. Roboflow and CVAT software were used to annotate the images using various classes including colony and cluster candidates. To ensure accurate AI model training, it was essential to label all visible objects in the images, including blurry colonies or clusters that were only in focus on a different plane.

Our proposed pipeline combines a YOLOv8 object detector with the BOTSort tracking algorithm to analyze colony formation in 3D cell cultures. The detector achieves good performance for the main classes of interest - colonies (mAP50 0.861) and clusters (mAP50 0.722). Lower performance on candidate objects and individual cells can be attributed to annotation inconsistencies or ambiguities rather than model limitations. Our tracker successfully maintains object identities across focal planes with IDF1 scores of 0.805 for colonies and 0.728 for clusters, with notably zero identity switches. This indicates that our adaptation of temporal tracking algorithms to the spatial domain of Z-stacks is effective. The system shows particular strength in tracking colonies, with on average 95% of colonies being either mostly or partially tracked.

Colony formation is not purely determined by the number of cells seeded; it also depends on the proximity and interactions between cells. This means that simply increasing the number of cells seeded does not necessarily lead to a proportionate increase in the number of colonies. Cooperative behavior disrupts the expected linear relationship between cell seeding and colony formation, making the clonogenic assay less predictable (34). To determine the optimal seeding density, various initial densities must be tested, which increases manual labor. Therefore, we tested the AI detection model to assess colony formation across different seeding densities to streamline the process and help identify the ideal conditions for accurate results. The preliminary results generated by the AI software served as a baseline for further downstream analysis.

To further validate the AI model, we performed IC_50_ as a part of a drug screening process. Traditionally, this is a cumbersome process, where the cells are treated with varying concentrations of drugs and monitored for colony formation and the colonies are counted manually at the endpoint to provide a relative colony count between treated and non-treated control samples. We utilized our automated microscopy and multiplexing approach to screen five different concentrations of three drugs, alongside a control chip. This innovative method allowed us to conduct a drug screening that differs from traditional techniques by enabling the tracking of individual cells to observe their dynamic responses to the drugs. Through this approach, we were able to capture the formation of colonies over time allowing us to capture crucial insights such as volume, size, and shape of the colonies, that are often overlooked when using conventional methods that only count colonies at the endpoint.

Our evaluation of the colony counting software against manual counts demonstrated Mean Absolute Errors (MAE) of 24 for single cells, 12 for clusters, and 1 for colonies. These findings align with the higher performance of the AI detection model in identifying colonies and lower performance in identifying single cells. The detection model occasionally misclassified larger single cells as clusters, leading to a slight overestimation in cluster counts. Additionally, colonies that were correctly classified at earlier time points were sometimes reclassified as clusters at later time points. The detection model also missed several single cells, leading to undercounts in this category. These discrepancies may reflect limitations in our training annotations and variations in manual counting. By minimizing these sources of human error such as creating a curated dataset that has been validated by multiple experts, the AI pipeline holds promise for standardized reproducible, and unbiased CFA.

Microfluidic devices are advanced platforms that are used as a way to deliver nutrients or compounds to cells closely mimicking physiological conditions (35–37). Although they have not fully replaced animal models, advancements in organoid or 3D cell culture are paving the way for more sophisticated *in-vitro* models to mimic drug treatments *in vivo*. Microfluidic devices enable continuous or intermittent perfusion of media containing nutrients, cytokines, or drugs at a constant flow rate.

In our system, we integrated a microfluidic device with automated nutrient and drug delivery, using Ibidi perfusion-based chips that allow cells to be embedded in collagen. Although we were able to successfully integrate the system, there are still parameters that need to be optimized to ensure the appropriate microenvironment for colony growth and formation. Our goal is to validate this approach in future studies. We expect to demonstrate that, once fully optimized and combined with AI-assisted colony-forming assays, *in vitro* microfluidics-based screening has the potential to revolutionize therapeutic approaches and advance personalized medicine.

The transition from traditional static assays to microfluidic systems presents significant opportunities but also several challenges that need to be addressed. One critical aspect is the establishment of microfluidic conditions, particularly in determining the appropriate drug concentrations and application frequency. Unlike traditional assays where the drug remains in a static environment, microfluidic systems continuously flush out drugs, which can lead to wastage and non-circulatory dynamics. This necessitates precise calibration to ensure that drug concentrations are effective while minimizing wastage.

Moreover, the AI detection model we currently employ is capable of generating raw data, but there is untapped potential in automating the subsequent analysis and data visualization steps. This advancement would not only streamline workflows but also enable more comprehensive, real-time insights into cell behavior during drug screening. Another advantage of software could be to predict the colony behavior and suggest an earlier or later endpoint to evaluate the effect of drugs or provide cell seeding density suggestions.

To date, our efforts have been focused on murine E2A-PBX1 B-ALL cells and cell lines, but an important future direction involves expanding the application to patient-derived samples. This would provide a more clinically relevant understanding of how therapies function in human cancer cells. Additionally, exploring combination therapies using this platform could uncover synergies between different drugs, which are often critical in complex diseases like cancer. Another area interesting for exploration is the biological underpinnings of why certain cells fail to form colonies. Understanding the heterogeneity in colony formation and why some cells behave differently could provide important insights into mechanisms of cancer relapse and resistance, offering new targets for therapeutic intervention.

## 5 Conclusions

In conclusion, transitioning from traditional colony formation assays to an AI-assisted, automated approach offers substantial advantages in terms of reducing manual labor, increasing accuracy, and enabling real-time monitoring. This study successfully demonstrated the feasibility of integrating automated time-lapse microscopy with AI-driven colony analysis for murine E2A-PBX1 B-ALL cells. By scaling down to a chip format and switching to collagen as a medium, we achieved comparable results to conventional methods while enhancing throughput and efficiency. The AI-assisted system not only automates colony counting but also provides dynamic insights into cell behavior during drug screening, capturing critical events like colony splitting. Overall, this innovative approach has the potential to significantly advance therapeutic development and personalized medicine, offering a more efficient, precise, and scalable method for evaluating treatment efficacy. Some limitations remain, particularly regarding annotation consistency and edge cases involving overlapping objects or ambiguous class definitions. In addition, cluster recognition and tracking could be further improved using additional subclasses or a more consistent single-cell detection, alleviating the need to combine smaller groups of individual cells into clusters. Nevertheless, the overall system demonstrates promising results for automated analysis of colony formation experiments.

## Data Availability

The raw data supporting the conclusions of this article will be made available by the authors, without undue reservation.

## References

[1] Shoemaker RH Wolpert-DeFilippes MK Kern DH Lieber MM Makuch RW Melnick NR. Application of a human tumor colony-forming assay to new drug screening. Cancer Res. (1985) 45:2145–53. 3986767

[2] Franken NAP Rodermond HM Stap J Haveman J Van Bree C. Clonogenic assay of cells *in vitro* . Nat Protoc. (2006) 1:2315–9. doi: 10.1038/nprot.2006.339 17406473

[3] Svozilova H Vojtova L Matulova J Bruknerova J Polakova V Radova L. *In vitro* culture of leukemic cells in collagen scaffolds and carboxymethyl cellulose-polyethylene glycol gel. PeerJ. (2024) 12:e18637. doi: 10.7717/peerj.18637 39655330 PMC11627079

[4] Astori G Malangone W Adami V Risso A Dorotea L Falasca E. A novel protocol that allows short-term stem cell expansion of both committed and pluripotent hematopoietic progenitor cells suitable for clinical use. Blood Cells Mol Dis. (2001) 27:715–24. doi: 10.1006/bcmd.2001.0439 11778655

[5] Mertelsmann R Lindemann A Oster W Gamm H Kolbe K Herrmann F. Hematopoietic growth factors in oncology. Cancer Detect Prev. (1990) 14:613–6. 2257558

[6] Li B Liu J Qu S Gale RP Song Z Xing R. Colony-forming unit cell (CFU-C) assays at diagnosis: CFU-G/M cluster predicts overall survival in myelodysplastic syndrome patients independently of IPSS-R. Oncotarget. (2016) 7:68023–32. doi: 10.18632/oncotarget.12105 PMC5356536 27655727

[7] Kaufman DS Hanson ET Lewis RL Auerbach R Thomson JA. Hematopoietic colony-forming cells derived from human embryonic stem cells. Proc Natl Acad Sci. (2001) 98:10716–21. doi: 10.1073/pnas.191362598 PMC58532 11535826

[8] Rajendran V Jain MV. *In vitro* tumorigenic assay: colony forming assay for cancer stem cells. In: Papaccio G Desiderio V, editors. Cancer Stem Cells. Springer New York, New York, NY (2018). p. 89–95. doi: 10.1007/978-1-4939-7401-6_8 28986889

[9] Katz D Ito E Lau KS Mocanu JD Bastianutto C Schimmer AD. Increased efficiency for performing colony formation assays in 96-well plates: novel applications to combination therapies and high-throughput screening. BioTechniques. (2008) 44:ix–xiv. doi: 10.2144/000112757 18422490

[10] Buch K Peters T Nawroth T Sänger M Schmidberger H Langguth P. Determination of cell survival after irradiation via clonogenic assay versus multiple MTT Assay–a comparative study. Radiat Oncol Lond Engl. (2012) 7:1. doi: 10.1186/1748-717X-7-1 PMC3274452 22214341

[11] Available online at: http://www.axionbiosystems.com/resources/application-note/quantifying-chemotoxicity-cancer-cell-colony-formation-using-live-cell? (Accessed September 17, 2024).

[12] Available online at: www.agilent.com/about/tektalk/en/newsletter-cancer-research.html (Accessed October 19, 2024).

[13] Available online at: https://www.agilent.com/cs/library/applications/automated-colony-formation-assay-5994-3403EN-agilent.pdf? (Accessed October 19, 2024).

[14] Zhang L. Machine learning for enumeration of cell colony forming units. Vis Comput Ind BioMed Art. (2022) 5:26. doi: 10.1186/s42492-022-00122-3 36334176 PMC9637067

[15] Carl SH Duempelmann L Shimada Y Bühler M. A fully automated deep learning pipeline for high-throughput colony segmentation and classification. Biol Open. (2020) 9:bio052936. doi: 10.1242/bio.052936 32487517 PMC7328007

[16] Siragusa M Dall’Olio S Fredericia PM Jensen M Groesser T. Cell colony counter called CoCoNut. Montazeri Aliabadi H, editor. PloS One. (2018) 13:e0205823. doi: 10.1371/journal.pone.0205823 30403680 PMC6221277

[17] Bray MA Vokes MS Carpenter AE. Using cellProfiler for automatic identification and measurement of biological objects in images. Curr Protoc Mol Biol. (2015) 109:14.17.1–14.17.13. doi: 10.1002/0471142727.mb1417s109 PMC4302752 25559103

[18] Guzmán C Bagga M Kaur A Westermarck J Abankwa D. ColonyArea: an imageJ plugin to automatically quantify colony formation in clonogenic assays. PloS One. (2014) 9:e92444. doi: 10.1371/journal.pone.0092444 24647355 PMC3960247

[19] Brzozowska B Gałecki M Tartas A Ginter J Kaźmierczak U Lundholm L. Freeware tool for analysing numbers and sizes of cell colonies. Radiat Environ Biophys. (2019) 58:109–17. doi: 10.1007/s00411-018-00772-z PMC6394662 30673853

[20] Shao Z Buchanan LB Zuanazzi D Khan YN Khan AR Prodger JL. Comparison between a deep-learning and a pixel-based approach for the automated quantification of HIV target cells in foreskin tissue. Sci Rep. (2024) 14:1985. doi: 10.1038/s41598-024-52613-3 38263439 PMC10806185

[21] Duque-Afonso J Lin CH Han K Wei MC Feng J Kurzer JH. E2A-PBX1 remodels oncogenic signaling networks in B-cell precursor acute lymphoid leukemia. Cancer Res. (2016) 76:6937–49. doi: 10.1158/0008-5472.CAN-16-1899 PMC5634812 27758892

[22] Duque-Afonso J Feng J Scherer F Lin CH Wong SHK Wang Z. Comparative genomics reveals multistep pathogenesis of E2A-PBX1 acute lymphoblastic leukemia. J Clin Invest. (2015) 125:3667–80. doi: 10.1172/JCI81158 PMC4588292 26301816

[23] Fry TJ Aplan PD. A robust *in vivo* model for B cell precursor acute lymphoblastic leukemia. J Clin Invest. (2015) 125:3427–9. doi: 10.1172/JCI83799 PMC4588242 26301807

[24] Tucker ER George S Angelini P Bruna A Chesler L. The promise of patient-derived preclinical models to accelerate the implementation of personalised medicine for children with neuroblastoma. J Pers Med. (2021) 11:248. doi: 10.3390/jpm11040248 33808071 PMC8065808

[25] Pasvolsky O Kebriaei P Shah BD Jabbour E Jain N. Chimeric antigen receptor T-cell therapy for adult B-cell acute lymphoblastic leukemia: state-of-the-(C)ART and the road ahead. Blood Adv. (2023) 7:3350–60. doi: 10.1182/bloodadvances.2022009462 PMC10345854 36912764

[26] Tang TCY Xu N Nordon R Haber M Micklethwaite K Dolnikov A. Donor T cells for CAR T cell therapy. biomark Res. (2022) 10:14. doi: 10.1186/s40364-022-00359-3 35365224 PMC8973942

[27] Raetz EA Teachey DT. T-cell acute lymphoblastic leukemia. Hematol Am Soc Hematol Educ Program. (2016) 1:580–8. doi: 10.1182/asheducation-2016.1.580 PMC6142501 27913532

[28] Dwyer B Nelson J Hansen T. Roboflow (Version 1.0) [Software]. (2024). Available at: https://roboflow.com/research#cite

[29] Aharon N Orfaig R Bobrovsky BZ. BoT-SORT: Robust Associations Multi-Pedestrian Tracking [Internet]. arXiv (2022). Available online at: http://arxiv.org/abs/2206.14651 (Accessed October 31, 2024).

[30] Jocher G Qiu J Chaurasia A. Ultralytics YOLO [Internet] (2023). Available online at: https://github.com/ultralytics/ultralytics (Accessed October 31, 2024).

[31] Akiba T Sano S Yanase T Ohta T Koyama M. (2019). Optuna: A next-generation hyperparameter optimization framework, in: Proceedings of the 25th ACM SIGKDD International Conference on Knowledge Discovery & Data Mining, . pp. 2623–31. New York, NY, USA: Association for Computing Machinery. doi: 10.1145/3292500.3330701

[32] CVAT.ai Corporation. Computer Vision Annotation Tool (CVAT) (2023). Available online at: https://github.com/cvat-ai/cvat.

[33] Heindl C. Pymotmetrics. Available online at: https://github.com/cheind/py-motmetrics (Accessed October 31, 2024).

[34] Brix N Samaga D Hennel R Gehr K Zitzelsberger H Lauber K. The clonogenic assay: robustness of plating efficiency-based analysis is strongly compromised by cellular cooperation. Radiat Oncol. (2020) 15:248. doi: 10.1186/s13014-020-01697-y 33121517 PMC7597001

[35] Petreus T Cadogan E Hughes G Smith A Pilla Reddy V Lau A. Tumour-on-chip microfluidic platform for assessment of drug pharmacokinetics and treatment response. Commun Biol. (2021) 4:1001. doi: 10.1038/s42003-021-02526-y 34429505 PMC8385015

[36] Zhai J Liu Y Ji W Huang X Wang P Li Y. Drug screening on digital microfluidics for cancer precision medicine. Nat Commun. (2024) 15:4363. doi: 10.1038/s41467-024-48616-3 38778087 PMC11111680

[37] Sun J Warden AR Ding X. Recent advances in microfluidics for drug screening. Biomicrofluidics. (2019) 13:061503. doi: 10.1063/1.5121200 31768197 PMC6870548

