# Supplementary material for: Leveraging automated time-lapse microscopy coupled with deep learning to automate colony forming assay
Source: Front Oncol. 2025 Feb 19;15:1520972. doi: 10.3389/fonc.2025.1520972 (PMC11879803; doi:10.3389/fonc.2025.1520972)
Supplement: Supplementary file 1 [file DataSheet1.docx]

**Supplementary**

**Supplementary 1: Demonstrative images of annotations of Z-stacks of time-lapse images using Roboflow.** Each cell or clump of cells in focus was labeled as a single cell (yellow), cluster (violet), compact colony (orange), or dispersed colony (aquamarine). Out-of-focus clumps were labeled as cluster candidates (red) or colony candidates (light blue). Black shadows represent clusters or colonies grown in different planes and the blurry background is due to collagen fibrils.


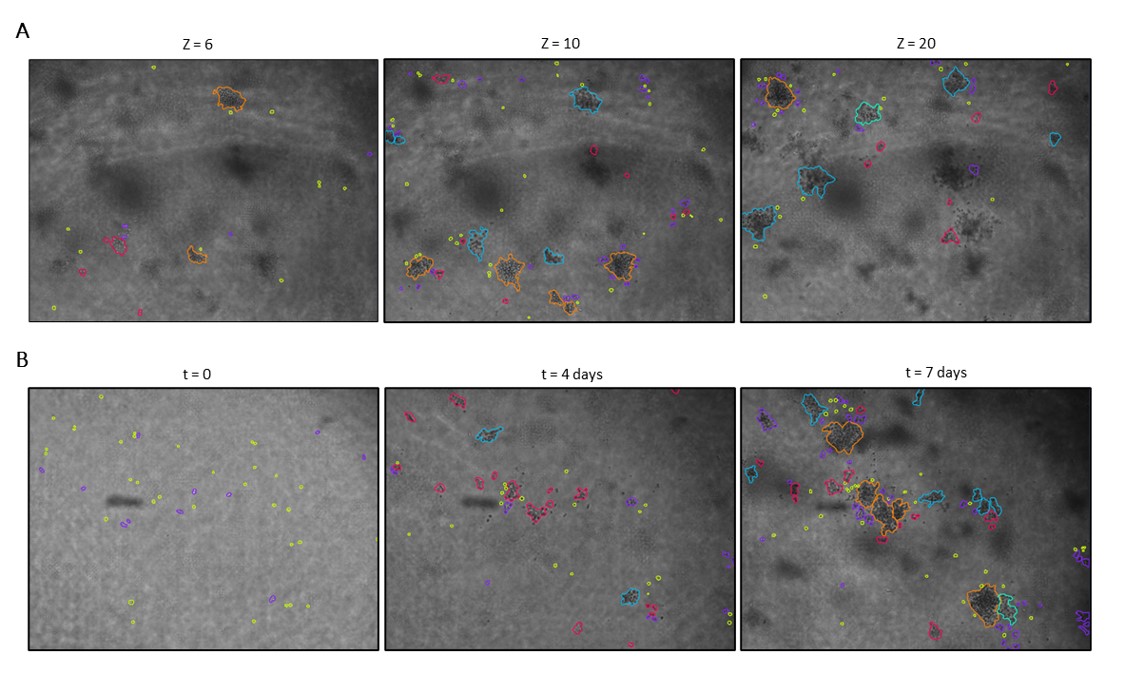


**Supplementary 2: Training Hyperparameters of YOLOv8-m.** The model was trained for 100 epochs with the 82 (246 including augmented) training images on a NVIDIA 3090 (24GB RAM) and a batch size of 8. Stochastic Gradient Descent was used as an optimizer and the inference resolution was doubled from the standard 640 pixels to 1280 pixels to improve detection on the large field of view of the microscopic images. Minimal runtime augmentations are used since the dataset already contains augmented images.

| {  "task": "detect",  "mode": "train",  "model": "yolov8m",  "epochs": 100,  "patience": 75,  "batch": 8,  "imgsz": 1280,  "cache": false,  "workers": 12,  "pretrained": true,  "optimizer": "SGD",  "seed": 0,  "deterministic": true,  "single_cls": false,  "rect": false,  "cos_lr": false,  "close_mosaic": 0,  "amp": true,  "dropout": 0.4,  "max_det": 3000,  "half": true,  "agnostic_nms": false,  "lr0": 0.005,  "lrf": 0.001,  "momentum": 0.937,  "weight_decay": 0.0005,  "warmup_epochs": 3.0,  "warmup_momentum": 0.8,  "warmup_bias_lr": 0.1, | "box": 6.5,  "cls": 1.0,  "dfl": 1.5,  "pose": 0,  "kobj": 0.0,  "label_smoothing": 0.1,  "nbs": 64,  "hsv_h": 0.0,  "hsv_s": 0.0,  "hsv_v": 0.0,  "degrees": 0.0,  "translate": 0.0,  "scale": 0.1,  "shear": 0.0,  "perspective": 0.0,  "flipud": 0.5,  "fliplr": 0.5,  "mosaic": 0.0,  "mixup": 0.2,  "copy_paste": 0.2  } |
| --- | --- |

**Supplementary 3: Final detection and tracking hyperparameters used for analysis of biological experiments.** Hyperparameters marked with #optimized were jointly optimized using the Optuna framework.

| detector:  agnostic_nms: true # optimized  augment: true # optimized  half: true  imgsz: 1280  max_det: 3000  min_confidence: 0.26523938455967816 # optimized  mode: detect  nms_max_iou: 0.33855380597078455 # optimized  single_cls: false  tracker:  with_reid: false  fuse_score: false  gmc_method: sparseOptFlow  fps: 7 # optimized  match_thresh: 0.8473897083832299 # optimized  new_track_thresh: 0.13615241040350018 # optimized  track_buffer: 7.0 # optimized  track_high_thresh: 0.13832210477043763 # optimized  track_low_thresh: 0.30762881544421017 # optimized  tracker_type: botsort |
| --- |
